# Trends in the Incidence of Disseminated Cryptococcosis in Japan: A Nationwide Observational Study, 2015–2021

**DOI:** 10.1007/s11046-023-00814-1

**Published:** 2024-01-17

**Authors:** Hidemasa Akazawa, Hideharu Hagiya, Toshihiro Koyama, Fumio Otsuka

**Affiliations:** 1 Department of General Medicine, Okayama University Graduate School of Medicine, Dentistry and Pharmaceutical Sciences, Okayama, 700-8558 Japan; 2 Department of Infectious Diseases, Okayama University Hospital, 2-5-1 Shikata-Cho, Kitaku, Okayama 700-8558 Japan; 3 Department of Health Data Science, Okayama University Graduate School of Medicine, Dentistry and Pharmaceutical Sciences, Okayama, 700-8558 Japan

**Keywords:** Disseminated cryptococcosis, Cryptococcal infection, Epidemiology, Trend analysis, Regionality

## Abstract

**Background:**

*Cryptococcus* species can cause severe disseminated infections in immunocompromised hosts. This study investigated the epidemiological features and trends in disseminated cryptococcosis in Japan.

**Methods:**

We used publicly available Infectious Diseases Weekly Reports to obtain data on the incidence of disseminated cryptococcosis in Japan from 2015 to 2021. Patient information, including age, sex, and regional and seasonal data, were extracted. The Joinpoint regression program was used to determine the age-adjusted incidence rate (AAR) per 100,000 population, annual percentage change (APC), and average APC (AAPC).

**Results:**

A total of 1047 cases of disseminated cryptococcosis were reported, of which those aged ≥ 70 years accounted for 68.8%. The AAR in men was significantly higher than that in women (median: 0.13 vs. 0.09: *p* = 0.0024). APC for the overall cases increased by 9.9% (95% confidence interval [95% CI] − 5.4–27.7) from 2015 to 2018 and then decreased by 3.3% (95% CI − 15.5–10.7) from 2018 to 2021. AAPC for the entire study period was 3.1% (95% CI − 1.5–8.0), indicating a possible increase in its number, although not statistically significant. In terms of regional distribution, the average AAR was highest in Shikoku District (0.17) and lowest in Hokkaido District (0.04). Northern Japan exhibited a significantly lower median AAR (median [interquartile range]: 0.06 [0.05, 0.08]) than the Eastern (0.12 [0.12, 0.13]), Western (0.11 [0.10, 0.13]), and Southern (0.14 [0.12, 0.15]) regions. No seasonal variation in incidence was observed.

**Conclusion:**

The prevalence of disseminated cryptococcosis has not increased in Japan. Geographically, the incidence is lower in Northern Japan. Further investigations that incorporate detailed clinical data are required.

**Supplementary Information:**

The online version contains supplementary material available at 10.1007/s11046-023-00814-1.

## Introduction

*Cryptococcus* species ubiquitously reside in the environment, such as soil and plants, and humans become infected with these opportunistic fungal pathogens through the inhalation of yeast, that specifically grows in bird feces [1]. *Cryptococcus neoformans* is the predominant pathogen responsible for human cryptococcal infections worldwide [2], causing progressive infections commonly in the lungs and central nervous system, particularly in immunocompromised hosts such as individuals with human immunodeficiency virus (HIV). In 2020, cryptococcal meningitis affected approximately 150,000 individuals with HIV worldwide, resulting in 112,000 deaths [3]. Recent studies have shown that patients without HIV, including organ transplant recipients, individuals with malignant diseases, those undergoing immunosuppressive therapy, and individuals with other potentially immunocompromising factors, are increasingly being reported to have cryptococcal infections [1, 4–8]. *Cryptococcus gattii* is a recently emerging strain that has the potential to cause meningitis in immunocompetent hosts, primarily in tropical and subtropical regions [9], as well as in Oceanian countries such as Australia and New Zealand [10]. In 2022, the World Health Organization published the Fungal Priority Pathogen List (FPPL) in response to the underdiagnosis of fungal infections and the issue of antifungal resistance [11]. In this document, *C. neoformans* and *C. gattii* were classified as the critical priority and medium priority groups, respectively, highlighting that cryptococcal diseases are among the most significant fungal infections worldwide, necessitating further research and clinical surveillance.

Disseminated cryptococcosis is a severe manifestation of cryptococcal infection that primarily affects immunocompromised individuals. It is defined as a case in which an organism is detected in aseptic clinical specimens such as blood and cerebrospinal fluid through either fungal culture or cryptococcal capsular antigen testing [12]. Due to advancements in medicine, the number of vulnerable individuals has increased, leading to the designation of disseminated cryptococcosis as a category V which is defined by the Infectious Diseases Act as notifiable infectious disease in 2015 to prevent outbreaks and spread in Japan [12]. Since then, to the best of our knowledge, no scientific analysis has been conducted using the accumulated data. Therefore, this study aimed to investigate the current epidemiology and trends in the incidence of disseminated cryptococcosis in Japan.

## Material and Methods

### Data Source

This retrospective observational analysis used publicly available data regarding the incidence of disseminated cryptococcosis in Japan between 2015 and 2021. The Infectious Diseases Weekly Reports of Japan, compiled by the National Institute of Infectious Diseases and based on the Act on the Prevention of Infectious Diseases and Medical Care for Patients with Infectious Diseases (Infectious Diseases Control Law), have provided information on the incidence of notifiable infectious diseases since November 2003. Since 2015, clinical doctors who diagnose the disease are required by law to notify the public health center within 7 days, as it is classified as a category V notifiable infectious disease. The data are then collected and made available to the public on the National Epidemiologic Surveillance of Infectious Diseases website [13]. As the law requires specific laboratory diagnoses for reporting, this study did not include clinically diagnosed cases. The required laboratory diagnoses are as follows: (i) microbiological isolation of the pathogen in clinical samples such as blood, ascites, pleural fluid, cerebrospinal fluid, and other aseptic samples; (ii) pathological detection of yeast with a capsular membrane; or (iii) a positive result for cryptococcal capsular antigen by latex agglutination.

### Data Processing and Statistical Analyses

The annual incidence of disseminated cryptococcosis was obtained from a publicly available website [13]. We retrieved the necessary data as Excel files and accessed the tally sheets for each fiscal year. These data files were used to extract the annual incidence rates by sex and age groups spanning ten years. Owing to the lack of detailed data for individuals aged 70 years or older, we treated the data for this age group as a single category (≥ 70 years). We calculated age-adjusted incidence rates (AARs) per 100,000 population using the distribution of 5-year age category in 2015 as the standard population. Vital statistics produced by the Japanese Ministry of Health, Labor, and Welfare were used to compile data on the Japanese population [14]. To determine and compare the AARs across regions, the data were stratified by reporting regions. The forty-seven prefectures were grouped into eight districts based on commonly used administrative classifications. The eight districts were categorized into the northern (Hokkaido and Tohoku), eastern (Kanto and Chubu), western (Kansai, Chugoku, and Shikoku), and southern (Kyushu) regions (Supplementary Table 1). Geographical software was used to illustrate the variations in AARs across each district (https://n.freemap.jp/tp/Japan). Additionally, we converted the weekly data into monthly data to assess the seasonality of disease onset as follows: winter (1st–8th and 48th–52nd or 53rd weeks), spring (9th–21st weeks), summer (22nd–34th weeks), and autumn (35th–47th weeks).

The Joinpoint Regression Program, version 5.0.2-May 25, 2023 (Statistical Research and Applications Branch, National Cancer Institute) was used to apply the joinpoint regression model by sex to estimate the trends in AARs [15]. The model can identify the year in which the major trends changed and assess the extent of the rise or drop. The annual percentage change (APC) was determined for each trend using generalized linear models under the assumption of a Poisson distribution for each joinpoint that exhibited a statistically significant change in the trend [16]. The average annual percentage change (AAPC) was estimated over the study period. Two-tailed 95% confidence intervals (CIs) were also calculated. EZR software, a graphical user interface for R 3.5.2, was used to perform the Kruskal–Wallis rank sum test and the Bonferroni-adjusted Mann–Whitney U test to compare the AARs among regions and seasons [17]. All reported *p* values were considered statistically significant when they were < 0.05.

## Results

### Numbers and AARs of the Disseminated Cryptococcosis Cases

**Fig. 1:**
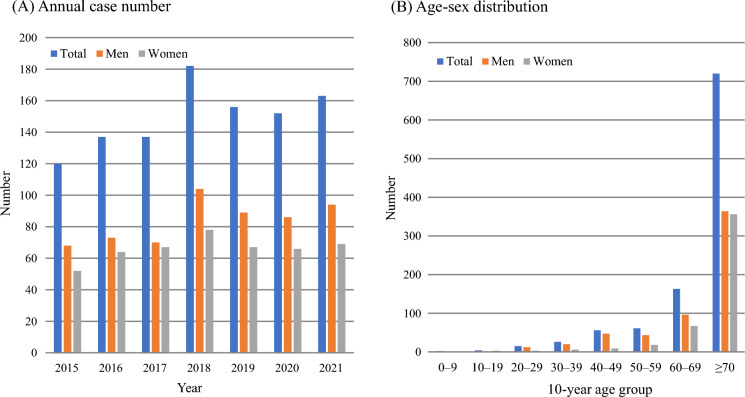
The annual case numbers (**A**) and its age-sex distribution (**B**) of notified disseminated cryptococcosis in Japan between 2015 and 2021

Between 2015 and 2021, 1047 cases (584 men and 463 women) of disseminated cryptococcosis were reported in Japan. The annual number of cases ranges from 120 to 182. The crude incidence rates increased with age, especially among those aged 60 years and above (Supplementary Fig. 1). The AARs per 100,000 population ranged between 0.09 and 0.12 (Supplementary Table 2). Each year, the number of cases in men was slightly higher than that in women (Fig. 1A). By age, patients aged 70 years or older accounted for most cases in both sexes, with 68.8% (720 cases) of the total cases. (Fig. 1B). The median [interquartile range] AAR in men (0.13 [0.12, 0.14]) was significantly higher than that in women (0.09 [0.09, 0.10]) (*p* = 0.0024) (Supplementary Fig. 2).

### Trend Analysis

We performed a trend analysis of the total AAR data by sex. Overall, the AAR first increased at the annual rate of 9.9% (95% CI − 5.4–27.7) from 2015 to 2018, and then decreased by 3.3% (95% CI − 15.5–10.7) from 2018 to 2021 (Table 1), resulting in AAPC of 3.1% (95% CI − 1.5–8.0) during the study period. In men, APC increased by 10.4% (95% CI − 5.6–29.2) between 2015 and 2018 and decreased by 1.3% (95% CI − 14.0–13.2) between 2018 and 2021, with 4.4% of AAPC (95% CI − 0.4–9.5). Among women, APC increased by 9.2% (95% CI − 6.3–27.2) in the first half, followed by a decreasing trend (− 6.0% [95% CI − 18.5–8.5]), without any increase or decrease in AAPC. Graphs of the joinpoint regression model are shown in Supplementary Fig. 3.

**Table 1:** Trend analysis in the age-adjusted rate per 100,000 population of disseminated cryptococcosis in Japan, 2015–2021

	Period 1		Period 2	
	Years	APC (95% CI)	Years	APC (95% CI)
Total	2015–2018	9.9% (− 5.4 to 27.7)	2018–2021	− 3.3% (− 15. 5 to 10.7)
Men	2015–2018	10.4% (− 5.6 to 29.2)	2018–2021	− 1.3% (− 14.0 to 13.2)
Women	2015–2018	9.2% (− 6.3 to 27.2)	2018–2021	− 6.0% (− 18.5 to 8.5)

*APC* annual percentage change, *CI* confidential interval

### Geographic Distribution

**Fig. 2:**
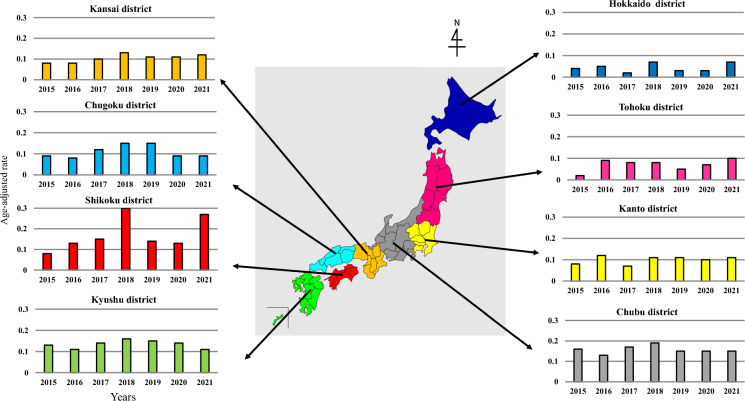
Geographic distribution of the age-adjusted incidence rates of disseminated cryptococcosis in Japan between 2015 and 2021

**Fig. 3:**
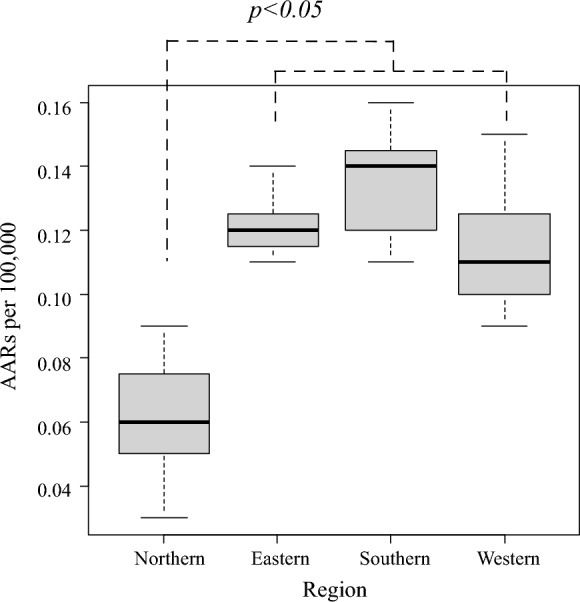
Regional comparison of the age-adjusted incidence rates (AAR) per 100,000 of disseminated cryptococcosis in Japan. The eight districts are categorized into northern (Hokkaido and Tohoku), eastern (Kanto and Chubu), western (Kansai, Chugoku, and Shikoku), and southern (Kyushu) regions. Northern Japan showed a significantly lower AAR than the other regions (vs. eastern, *p* = 0.012; vs. southern, *p* = 0.013; and vs. western,* p* = 0.019), and the Kruskal–Wallis test, with *p*-value adjustment using the Bonferroni method, was conducted to compare the data among the four regions

The annual AARs per 100,000 population in the eight Japanese districts are demonstrated in Fig. 2. The average AAR [95% CI] in each district during the study period was as follows: Hokkaido (0.04 [0.03, 0.06]), Tohoku (0.07 [0.04, 0.10]), Kanto (0.1 [0.08, 0.12]), Chubu (0.16 [0.14, 0.17]), Kansai (0.10 [0.09, 0.12]), Chugoku (0.11 [0.08, 0.14]), Shikoku (0.17 [0.10, 0.25]), and Kyushu (0.13 [0.12, 0.15]). The eight districts were further grouped into four regions according to their location, and the median annual AARs were compared. The median AAR [interquartile range] in the northern region (0.06 [0.05, 0.08]) was significantly lower than that in the other three regions (eastern: 0.12 [0.12, 0.13], western: 0.11 [0.10, 0.13], southern: 0.14 [0.12, 0.15]) (Fig. 3). A similar result was observed when stratifying the data for those aged 70 years or older (Supplementary Fig. 4).

### Seasonality

**Fig. 4:**
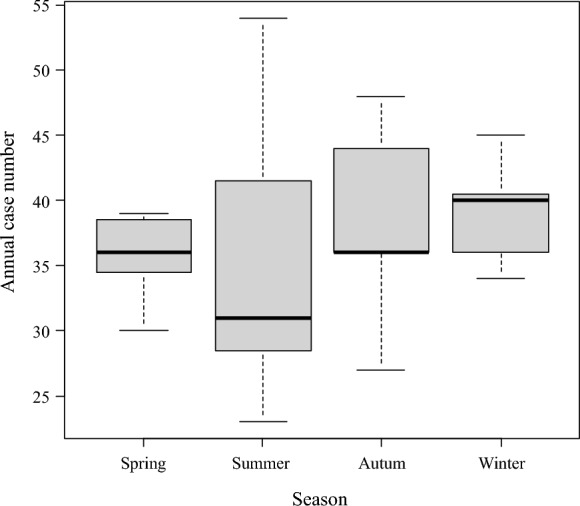
Seasonal comparison of the incidence of disseminated cryptococcosis in Japan. No seasonal differences were observed (*p* = 0.72 [Kruskal–Wallis test])

Finally, the incidence of disseminated cryptococcosis was compared across the four seasons (Fig. 4). Median numbers [interquartile range] in spring, summer, autumn, and winter were 36 [34.5–38.5], 31 [28.5–41.5], 36 [36.0–44.0], and 40 [36.0–40.5], respectively. The results of the Kruskal–Wallis test suggested that there were no seasonal biases (*p* = 0.72).

## Discussion

To the best of our knowledge, this is the first endeavor to elucidate the current epidemiology of disseminated cryptococcosis in Japan using multifaceted approaches and analysis. The findings underscored that (i) disseminated cryptococcosis predominantly affects the elderly population, and (ii) significantly more common in men, (iii) no discernible upward or downward trend was observed, and (iv) the incidence in Northern Japan was approximately half that in the other regions.

Approximately 70% of the reported cases were in patients aged 70 years or older, indicating a significant risk to the geriatric population in Japan. A recent study unequivocally demonstrated the high clinical burden of cryptococcosis in HIV-positive individuals worldwide [3]. Conversely, a recent multicenter cohort study indicated that 90% of cryptococcosis cases involved patients without HIV [5], implying a shift in the epidemiology of the disease in developed countries. This can be attributed to the reduction in uncontrolled HIV due to the enhanced availability of highly effective antiretroviral therapy, as well as the increase in immunocompromised patients due to other factors. However, to the best of our knowledge, only a few studies have investigated the epidemiology and clinical characteristics of disseminated cryptococcosis in HIV-negative populations [8, 18]. Apart from the physiological decline in immune function [19], a higher prevalence of various comorbidities such as malignancies [20], diabetes [21], and chronic organ diseases [22] provides an explanatory link to the increasing susceptibility of older individuals to cryptococcosis. In this aging world, there is a growing concern that the incidence of disseminated cryptococcosis, a potentially life-threatening disease, will rise in other countries.

In general, the occurrence of disseminated cryptococcosis exhibited a notably higher frequency in men. This phenomenon may be attributed to disparities in the prevalence of immunocompromising factors between the sexes, including conditions such as HIV, malignancies, and diabetes [20, 23, 24]. Furthermore, sex differences in immune responses related to viral infections have recently garnered attention, supposedly involving a range of cytokines, chemokines, immune cells, and sex hormones [25]. These differences could potentially elucidate the vulnerability of men to various infectious diseases. Nevertheless, the applicability of these findings to fungal infections requires investigation in future research.

There has been no particular trend in the AARs of disseminated cryptococcosis since 2015 when the disease was designated as a notifiable disease in Japan. The total male and female data exhibited upward trends until 2018, albeit without a clear statistical significance. However, they subsequently exhibited a declining trend until the end of the study period. As far as we were concerned, there was no outbreak event or particular episodes in 2018. Given the global pandemic of the novel coronavirus disease 2019, it is possible that the disease has been underreported, as has been observed in other infectious diseases [26, 27]. With advancements in medical technology, the number of immunocompromised individuals at an elevated risk of developing disseminated cryptococcosis will continue to increase. Vigilant observations are necessary to comprehend the future epidemiology of this fatal opportunistic disease in developed countries.

Our data revealed a geographically biased distribution of disseminated cryptococcosis in Japan. AARs in the Hokkaido and Tohoku districts were comparatively lower at 0.04 and 0.07 per 100,000 population, respectively, with the northern region exhibiting a significantly lower AAR than the other regions. A clear elucidation of the geographic differences is not currently available; however, it is possible that differences in the prevalence of Cryptococcus species and populations of immunocompromised individuals may be contributing factors. As has been reported internationally, no seasonality has been demonstrated in the incidence of this disease. This finding suggests that physicians should be aware of cryptic diseases throughout the year.

Several limitations of this study should be acknowledged. First, underdiagnosis is a potentially significant issue owing to the nature of data collection and techniques. Given that cryptococcosis is a relatively rare disease, physicians may inadvertently overlook it in daily medical practice. Secondly, due to the absence of clinical data, we were unable to assess the data from a clinical standpoint. The underlying medical conditions of the patients, notably HIV status and immunocompromising factors, are critical but were not accessible in this study. Thirdly, our dataset lacked comprehensive fungal identification, primarily due to the unavailability of such information in the open database. It is noteworthy that clinical characteristics and outcomes differ significantly between *C. neoformans* and *C. gattii*, and ideally, this should have been integrated into the analysis. Fourth, the present study did not examine patient outcomes, such as mortality. Fifth, data beyond 2022 has not been incorporated. The reason for this omission is the absence of an official announcement regarding detailed data. Currently, the only information ascertainable from the weekly reports pertains to the region and season of the year, with no data available concerning age and gender. Despite these limitations, we believe that the current data should be shared with medical professionals and researchers to enhance their understanding of the epidemiological characteristics of this disease for future reference.

Collectively, there is no clear evidence of the increasing prevalence of disseminated cryptococcosis in Japan. However, in this era of population aging and advances in medicine, the population at risk of disease is expected to increase. Disease incidence appears to vary geographically among the different regions in Japan; however, a more detailed analysis incorporating the clinical characteristics of patients is necessary to reach a definitive conclusion. Notably, our data focused solely on disseminated cases, and the epidemiology of cryptococcosis as a whole, including pulmonary cryptococcosis, requires further studies.

### Supplementary Information

Below is the link to the electronic supplementary material.

Supplementary file1 (PPTX 168 kb)

Supplementary file2 (DOCX 33 kb)

## Data Availability

The datasets generated and analyzed in the current study are available from the corresponding author on request.
