# Supplementary material for: Trends in the Incidence of Disseminated Cryptococcosis in Japan: A Nationwide Observational Study, 2015–2021
Source: Mycopathologia. 2024 Jan 17;189(1):8. doi: 10.1007/s11046-023-00814-1 (PMC10794261; doi:10.1007/s11046-023-00814-1)
Supplement: Supplementary file 1 — Supplementary file1 (PPTX 168 kb) [file 11046_2023_814_MOESM1_ESM.pptx]

## Slide 1
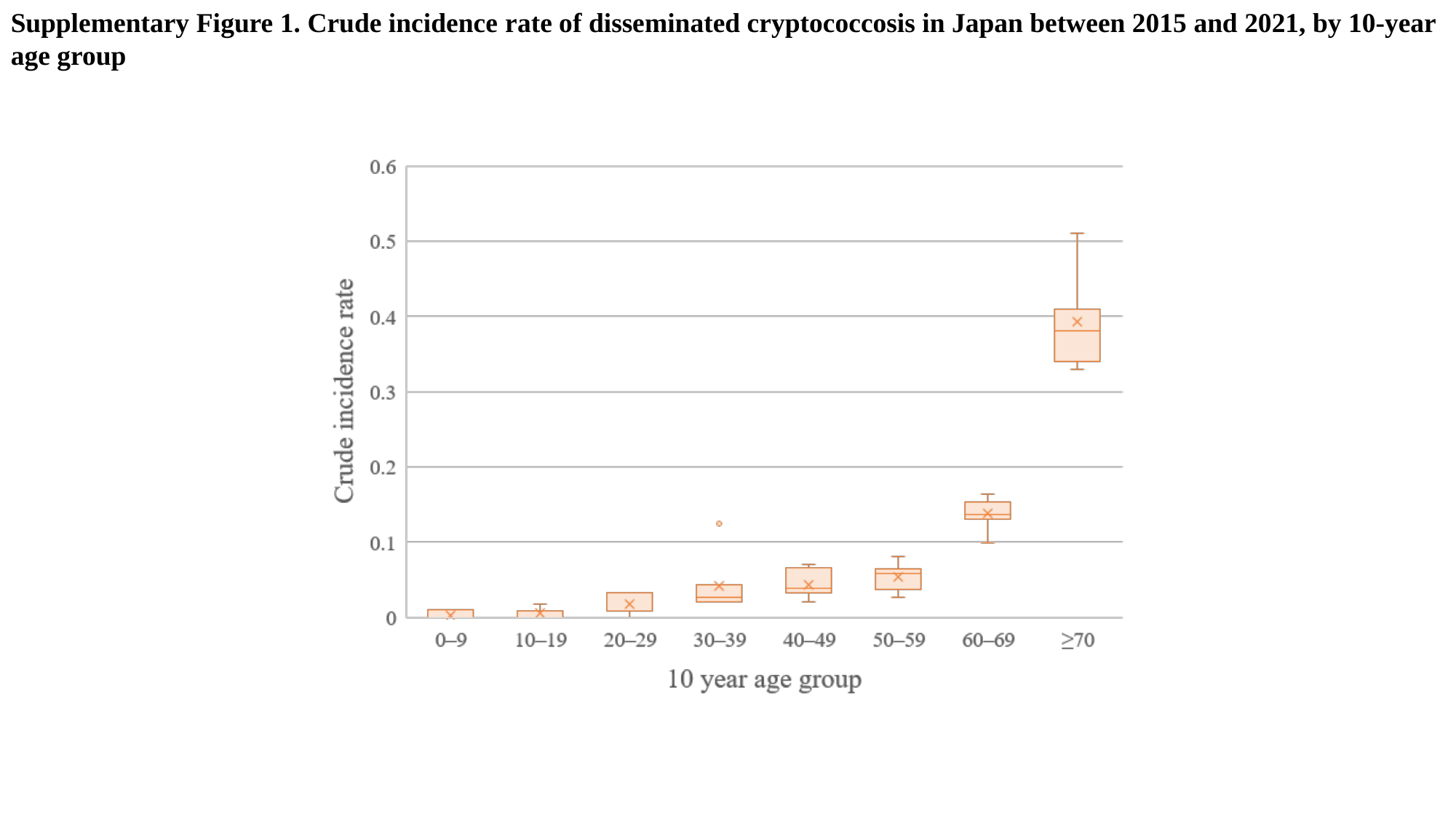

Supplementary Figure 1. Crude incidence rate of disseminated cryptococcosis in Japan between 2015 and 2021, by 10-year age group

## Slide 2
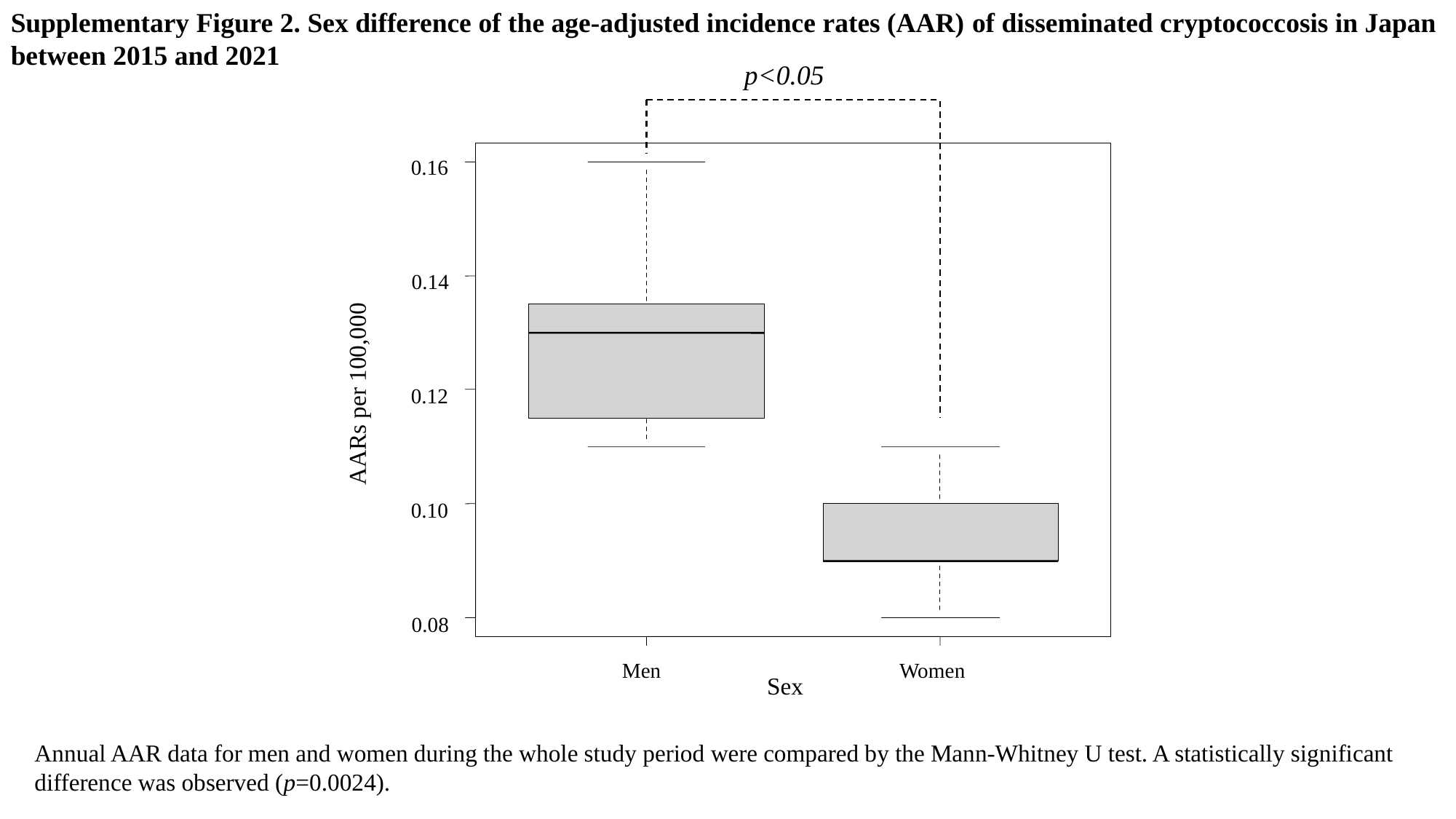

Supplementary Figure 2. Sex difference of the age-adjusted incidence rates (AAR) of disseminated cryptococcosis in Japan between 2015 and 2021
p<0.05
0.16
0.14
AARs per 100,000
0.12
0.10
0.08
Men
Women
Sex
Annual AAR data for men and women during the whole study period were compared by the Mann-Whitney U test. A statistically significant difference was observed (p=0.0024).

## Slide 3
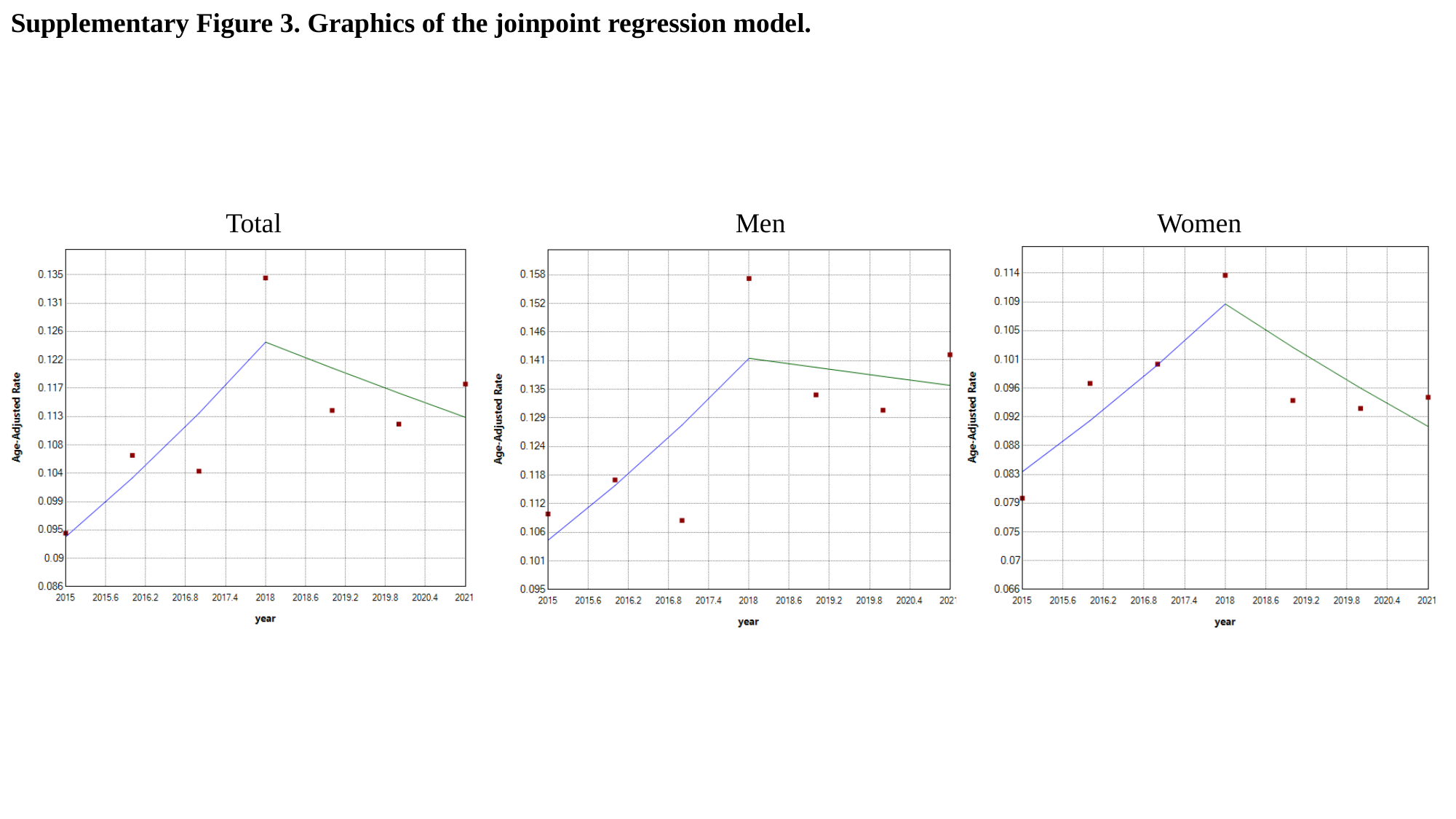

Supplementary Figure 3. Graphics of the joinpoint regression model.
Total
Men
Women

## Slide 4
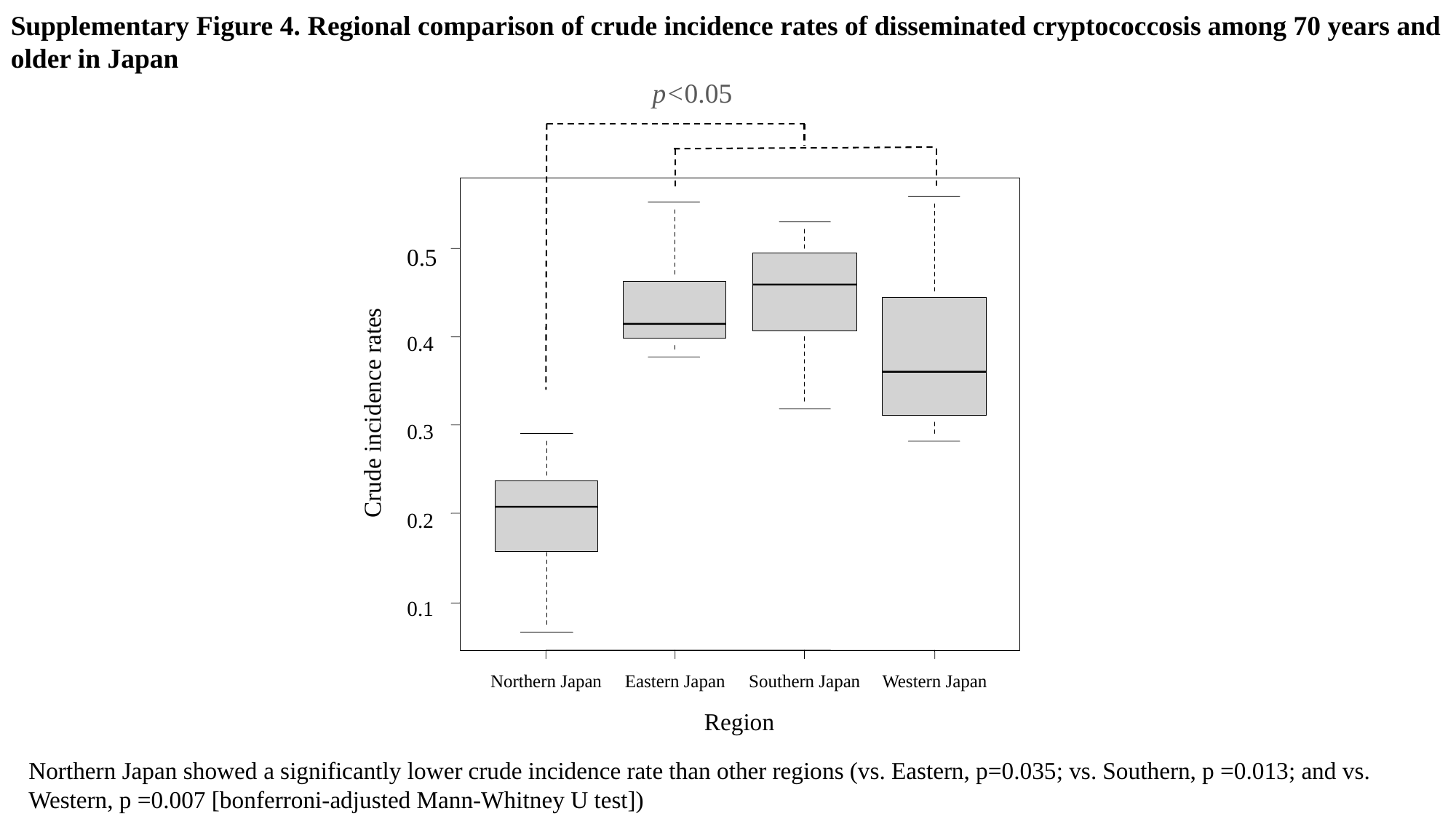

Supplementary Figure 4. Regional comparison of crude incidence rates of disseminated cryptococcosis among 70 years and older in Japan
p<0.05
0.5
0.4
Crude incidence rates
0.3
0.2
0.1
Northern Japan
Eastern Japan
Southern Japan
Western Japan
Region
Northern Japan showed a significantly lower crude incidence rate than other regions (vs. Eastern, p=0.035; vs. Southern, p =0.013; and vs. Western, p =0.007 [bonferroni-adjusted Mann-Whitney U test])
