# Supplementary material for: Trends in the Incidence of Disseminated Cryptococcosis in Japan: A Nationwide Observational Study, 2015–2021
Source: Mycopathologia. 2024 Jan 17;189(1):8. doi: 10.1007/s11046-023-00814-1 (PMC10794261; doi:10.1007/s11046-023-00814-1)
Supplement: Supplementary file 2 — Supplementary file2 (DOCX 33 kb) [file 11046_2023_814_MOESM2_ESM.docx]

**Supplementary Table 1. The groups and regions of 47 prefectures in Japan.**

| **Prefecture** | **District** | **Region** |
| --- | --- | --- |
| Hokkaido | Hokkaido district | Northern Japan |
| Aomori | Tohoku district |  |
| Iwate |  |  |
| Miyagi |  |  |
| Akita |  |  |
| Yamagata |  |  |
| Fukushima |  |  |
| Ibaraki | Kanto district | Eastern Japan |
| Tochigi |  |  |
| Gunma |  |  |
| Saitama |  |  |
| Chiba |  |  |
| Tokyo |  |  |
| Kanagawa |  |  |
| Niigata | Chubu district |  |
| Toyama |  |  |
| Ishikawa |  |  |
| Fukui |  |  |
| Yamanashi |  |  |
| Nagano |  |  |
| Gifu |  |  |
| Shizuoka |  |  |
| Aichi |  |  |
| Mie | Kansai district | Western Japan |
| Shiga |  |  |
| Kyoto |  |  |
| Osaka |  |  |
| Hyogo |  |  |
| Nara |  |  |
| Wakayama |  |  |
| Tottori | Chugoku district |  |
| Shimane |  |  |
| Okayama |  |  |
| Hiroshima |  |  |
| Yamaguchi |  |  |
| Tokushima | Shikoku district |  |
| Kagawa |  |  |
| Ehime |  |  |
| Kochi |  |  |
| Fukuoka | Kyushu district | Southern Japan |
| Saga |  |  |
| Nagasaki |  |  |
| Kumamoto |  |  |
| Oita |  |  |
| Miyazaki |  |  |
| Kagoshima |  |  |
| Okinawa |  |  |

**Supplementary Table 2. Case numbers and age-adjusted rate per 100,000 population of the incidence of disseminated cryptococcosis in Japan, 2015-2021**

|  |  |  | **Age-adjusted rate of the incidence per 100,000 population** | | |
| --- | --- | --- | --- | --- | --- |
| Year | **Case No.** |  | **Total** | **Men** | **Women** |
| 2015 | 120 |  | 0.09 | 0.10 | 0.08 |
| 2016 | 137 |  | 0.10 | 0.12 | 0.09 |
| 2017 | 137 |  | 0.11 | 0.13 | 0.10 |
| 2018 | 182 |  | 0.12 | 0.14 | 0.11 |
| 2019 | 156 |  | 0.12 | 0.14 | 0.10 |
| 2020 | 152 |  | 0.12 | 0.14 | 0.10 |
| 2021 | 163 |  | 0.11 | 0.14 | 0.09 |

Age-adjusted rates were calculated using data in 2015 as a standard population.
